# Plant chlorophyll fluorescence: active and passive measurements at canopy and leaf scales with different nitrogen treatments

**DOI:** 10.1093/jxb/erv456

**Published:** 2015-10-19

**Authors:** M. Pilar Cendrero-Mateo, M. Susan Moran, Shirley A. Papuga, K.R. Thorp, L. Alonso, J. Moreno, G. Ponce-Campos, U. Rascher, G. Wang

**Affiliations:** ^1^Soil Water and Environmental Science, The University of Arizona, 1177 East Fourth Street, Tucson 85721, USA; ^2^USDA Southwest Watershed Research Center, 2000 East Allen Road, Tucson, AZ 85719, USA; ^3^School of Natural Resources, The University of Arizona, 325 Biosciences East, Tucson, AZ 85721, USA; ^4^USDA Arid-Land Agricultural Research Center, 21881 North Cardon Lane, Maricopa, AZ 85138, USA; ^5^Image Processing Laboratory (IPL), Universitat de Valencia Catedratico A. Escardino - 46980 Paterna, Valencia, Spain; ^6^Institute of Bio- and Geosciences, Forschungszentrum Jülich GmbH, Leo-Brandt-Str., 52425 Jülich, Germany; ^7^Bridgestone Americas Agricultural Operations, 4140W. Harmon Rd., Eloy, AZ 85131, USA

**Keywords:** FLD, Fluowat, PAM, nitrogen, chlorophyll content.

## Abstract

Most studies assessing chlorophyll fluorescence (ChlF) have examined leaf responses to environmental stress conditions using active techniques. Alternatively, passive techniques are able to measure ChlF at both leaf and canopy scales. However, the measurement principles of both techniques are different, and only a few datasets concerning the relationships between them are reported in the literature. In this study, we investigated the potential for interchanging ChlF measurements using active techniques with passive measurements at different temporal and spatial scales. The ultimate objective was to determine the limits within which active and passive techniques are comparable. The results presented in this study showed that active and passive measurements were highly correlated over the growing season across nitrogen treatments at both canopy and leaf-average scale. At the single-leaf scale, the seasonal relation between techniques was weaker, but still significant. The variability within single-leaf measurements was largely related to leaf heterogeneity associated with variations in CO_2_ assimilation and stomatal conductance, and less so to variations in leaf chlorophyll content, leaf size or measurement inputs (e.g. light reflected and emitted by the leaf and illumination conditions and leaf spectrum). This uncertainty was exacerbated when single-leaf analysis was limited to a particular day rather than the entire season. We concluded that daily measurements of active and passive ChlF at the single-leaf scale are not comparable. However, canopy and leaf-average active measurements can be used to better understand the daily and seasonal behaviour of passive ChlF measurements. In turn, this can be used to better estimate plant photosynthetic capacity and therefore to provide improved information for crop management.

## Highlight

We studied for the first time the temporal and spatial limits within which active and passive chlorophyll fluorescence measurements are comparable.

## Introduction

**Fig. 1.**
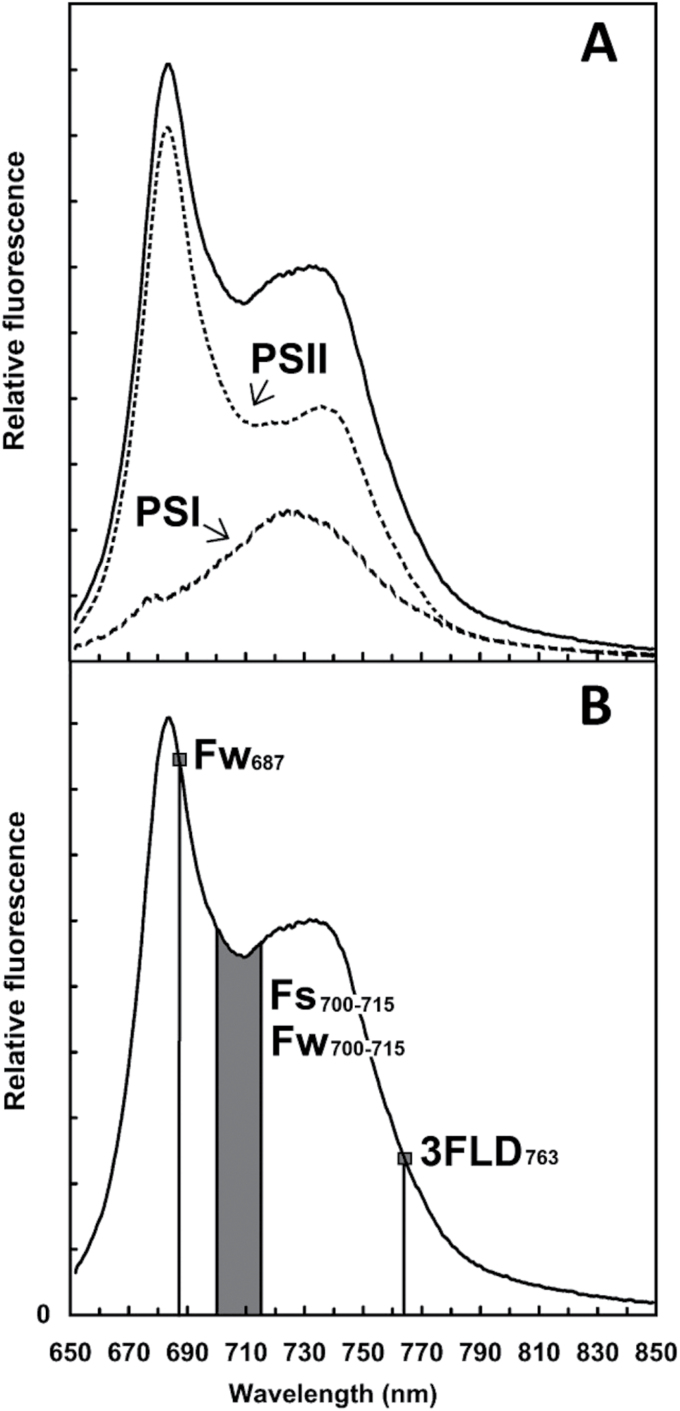
(A) Chlorophyll fluorescence spectrum at F_o_ where the red band is mainly emitted from photosystem II (PSII) and the far-red band from photosystem I (PSI). (B) Chlorophyll fluorescence spectrum measurements. Active technique, *F_s700–715_*, grey highlighted area between 700 and 715 nm. Passive technique, Fluowat (*Fw_687_* and grey highlighted area between 700 and 715 nm, *Fw_700–715_*,), and FLD (*3FLD_763_*). Reprinted with permission from Franck *et al.* (2002).

One promising approach for obtaining global estimates of plant photosynthesis is the use of chlorophyll fluorescence (ChlF). ChlF are photons of red and far-red light (Fig. 1A) that are emitted by chlorophyll *a* pigments nanoseconds after light absorption (Porcar-Castell *et al.*, 2014). The principle underlying the use of ChlF as an indicator of plant physiological status is relatively straightforward. Absorbed light energy excites chlorophyll molecules and de-excitation of this energy is mainly attained through three competing processes: photosynthesis, radiative loss of photons or ChlF, and non-radiative thermal energy dissipation (NPQ). As these three energy dissipation processes compete for excitation energy, changes in one process (e.g. photosynthesis) will affect the other two. Hence, by measuring ChlF, we can derive information on NPQ and photosynthesis. (Maxwell and Johnson, 2000; Porcar-Castell *et al.*, 2014).

The use of ChlF to detect plant stress at leaf and canopy scale has been demonstrated in numerous studies. Flexas *et al.* (2002) and Zarco-Tejada *et al.* (2013) reported that ChlF was a good indicator of water deficit in vineyard plants. Additionally, Schächtl *et al.* (2005) and Corp *et al.* (2009) showed that ChlF was a good indicator of nitrogen status in corn and wheat plants, respectively. These experiments were used different measurement techniques: active techniques for leaf-scale and passive techniques for canopy-scale measurements.

Major developments in the instrumentation for measuring ChlF have been made in the last decade. Active techniques, based on pulse-amplitude modulation (PAM, Bolhar-Nordenkampf *et al.*, 1989) or the laser-induced fluorescence transient method (LIFT, Kolber *et al.*, 2005; Roland Pieruschka, 2010) allow the estimation of the relative variation in the steady-state chlorophyll fluorescence (F_s_) in light-adapted plants, which is related to actual plant photosynthesis efficiency. In addition, active-based instruments also allow the calculation of other ChlF parameters, such as minimal fluorescence (F_o_ − emitted in the absence of photosynthetic light) and maximal fluorescence (F_m_ − emitted after applying a short saturating light pulse). Knowledge of these parameters together with absorbed photosynthetic active radiation (aPAR) is important to compute the electron transport for photosynthesis. On the one hand, because active techniques use an artificial light to excite the leaf, they are limited in application to spatial scales ranging from several centimetres to some metres (Kolber *et al.*, 2005; Amoros-Lopez *et al.*, 2008). Alternatively, passive techniques retrieve the ChlF emission from the solar irradiance and the radiance emitted by vegetation by using the absorption bands in surface solar irradiance (termed sun-induced fluorescence, SIF). The Fraunhofer Line Discrimination (FLD) principle (Plascyk, 1975; Plascyk and Grabriel, 1975) is the most widely method used to retrieve ChlF emission using passive techniques. These techniques allow the estimation of the absolute variation in the steady-state chlorophyll fluorescence at leaf, canopy and regional scale, but do not allow the estimation of F_o_ and F_m_.

Both PAM fluorometers and the FLD provide information about ChlF. However their physical measuring principles differ, where (i) PAM uses a weak and constantly modulated light to induced fluorescence in contrast to FLD, which measures the total fluorescence emitted in response to solar illumination, and (ii) PAM measures ChlF in a broad band (700−715nm in the case of Licor 6400), while FLD measures ChlF in a narrower band of 1−2nm at 687nm O_2_B and 763nm O_2_A (Amoros-Lopez *et al.*, 2008). Despite these differences, active measurements have been used to better understand the mechanisms that control SIF and its relationship with photosynthesis and plant physiological status (Anon., 2013). However, only a few datasets concerning the relationship between these two techniques have been reported in the literature.

At canopy scale, Zarco-Tejada *et al.* (2013) investigated active and passive ChlF changes in vineyards under water deficit. Perez-Priego *et al.* (2005) and Rascher *et al.* (2009) correlated diurnal changes of active and passive ChlF in olive orchards and corn fields, respectively, and Louis *et al.* (2005) compared active and passive measurements in a boreal forest during spring recovery. In these experiments, active measurements were performed at leaf scale and passive measurements at canopy scale. Therefore, in order to compare both datasets, a representative number of leaves were measured using the active technique and then averaged to a unique value for the canopy. Zarco-Tejada *et al.* (2013) compared active (leaf) and passive (airborne) measurements taken in 2011 and 2010 during two consecutive days. Measurements were taken at midday (11:00–13:00h) in several study plots under different water treatments. Under these conditions, a weak but significant correlation between F_s_ and SIF was found (*R* ^2^=0.40, *P*<0.01, Zarco-Tejada *et al.* 2013). Additionally, diurnal cycle studies performed by Perez-Priego *et al.* (2005) and Rascher *et al.* (2009) analysed how ChlF changes with light stress. These measurements were made in a single day in one field plot under the same treatment (well-watered or water limited). Under these conditions, a positive correlation between active and passive techniques was found (*R* ^2^=0.6−0.9, Perez-Priego *et al.*, 2005). Finally, Louis *et al.* (2005) reported a similar trend when seasonal passive and active measurements were compared, though a direct correlation between both techniques was not presented.

At leaf scale, Moya *et al.* (2004) compared leaf active and passive measurements during a dark-to-light transition in a single bean leaf attached to the plant. In this study, dark-adapted leaves were exposed to bright sunlight inducing strong variations in ChlF, which were simultaneously recorded by the two instruments. When plotting the passive fluorescence signal versus the active fluorescence signal, a very high linear correlation coefficient was obtained (*R* ^2^>0.99). Importantly, the fit extrapolated to a zero value, which means that both signals were proportional.

Previous studies have reported that active and passive techniques at both canopy and leaf scale showed a strong correlation when light was the limiting factor and measurements were conducted in one day in a single plot under the same treatment (Perez-Priego *et al.*, 2005; Rascher *et al.*, 2009; Moya *et al.*, 2004). However, when measurements were taken in multiple days through different study plots and treatments, no correlation was presented (Louis *et al.*, 2005) or a weak correlation was found (Zarco-Tejada *et al.*, 2013).

Because of this discrepancy in the results of previous studies, combined measurements of ChlF based on PAM and FLD techniques are needed at daily to seasonal time scales and at leaf to canopy spatial resolutions, to define the limits where it is possible to extrapolate the knowledge acquired using active techniques to passive techniques. In this study, we compared ChlF measurements using active techniques with SIF measurements at different temporal and spatial scales. The ultimate objective was to determine the temporal and spatial limits within which active and passive techniques are comparable. In turn, this can be used to better understand the spatio-temporal variation in SIF measured from a tower (FUSION, NASA/GSFC) or aircraft (Rascher *et al.*, 2013) and will support the potential launch of the FLuorescence EXplorer (FLEX) (European Space Agency, 2008).

## Materials and methods

### Field experiment

A wheat experiment was conducted at the University of Arizona’s Maricopa Agricultural Center (MAC) near Maricopa, Arizona (33.067547 °N, 111.97146 °W) over the winter of 2011/12. A split-plot design with three replicates of *Orita* wheat cultivar under three nitrogen fertilizer applications rates was used for the experiment. Wheat was planted on 9 December 2011 with a row spacing of 19.05cm. A Sudan grass cover crop was grown in the summer of 2010 to remove excess nitrate from the soil. The entire experimental area was flood irrigated to avoid water deficits. The total depth of irrigation water was 692mm, applied in nine irrigation events from 9 December 2011 to 30 April 2012. Precipitation amounted to 45mm over the growing season. The soil texture at the site was predominantly sandy loam and sandy clay loam, as determined by textural analysis of soil samples collected after planting. After two months of plant growth, ChlF measurements at both canopy and leaf scale were taken once a week from 24 February to 27 April 2012, for a total of 9 d. At leaf level, three samples per nitrogen treatment, replicate and day were taken (*n*=243). The following active and passive techniques were used to measure ChlF.

### Active technique: modulated artificial light-induced chlorophyll fluorescence

Active measurements were made at leaf scale with the Licor 6 400–40 leaf chamber fluorometer, referred to here as LCF (Li-COR Biosciences, Lincoln, NE, USA). Active techniques use modulated artificial light and a detector to measure relative F_s_ in arbitrary units (au). F_s_ at LCF is excited by a very brief and weak pulse of red light (centre wavelength ~630nm). The incident radiation causes an oscillation in fluorescence with a small amplitude (<1 μmol photons m^-2^s^-1^); but because it is regular, it can be detected and demodulated by circuitry in the LCF. The detector is filtered to measure radiation between 700 and 715nm (F_s700-715_, Fig. 1B) and the demodulated signal sent to the LCF is proportional to the amplitude of the detected fluorescence oscillation (LCF manual, Li-COR Biosciences, Lincoln, NE, USA) (Table 1).

**Table 1.** Chlorophyll fluorescence (ChlF) active and passive measurement techniques (adapted from Amoros-Lopez *et al.*, 2008)

	**Active technique**	**Passive technique**	
Approach	PAM (Licor 6 400)	FLD (3FLD)	Filtered illumination (Fluowat)
Measuring light	Modulated artificial red LEDs	Sunlight	Filtered artificial or sunlight
ChlF spectrum measurement wavelength	700−715 nm	687nm (O_2_-B band) 763nm (O_2_-A band)	Whole ChlF spectrum
Main contributors to ChlF (Fig. 1A)	PSII & PSI	PSII & PSI	PSII & PSI
Target distance	cm/ground/airborne observation	cm/ground/airborne/satellite observation	cm
Measurement type	Relative fluorescence yield	ChlF emission peaks in radiance	ChlF emission spectrum in radiance

The ChlF emission spectrum of a green leaf taken at ambient temperatures is characterized by two main emission peaks at 690 and 740nm (Fig. 1). The relative height and wavelength position of the chlorophyll fluorescence emission peaks depends upon the chlorophyll content of the leaves and Photosystem I (PSI) and Photosystem II (PSII) efficiency (Lichtenthaler and Rinderle, 1988). At ambient temperature, ChlF is mainly emitted by PSII and only a small contribution is emitted from PSI. The latter consists primarily of ﬂuorescence light in the spectral range above 710nm (Buschmann, 2007).

Importantly, at 700−715nm, both PSI and PSII contribute to the ChlF measured by LI-6 400 (Fig. 1A). In addition, photosynthetically active radiation (PAR) and gas exchange parameters (photosynthesis and stomatal conductance to water vapour) were measured in parallel with F_s_ by the LCF. Gas exchange parameters were used to estimate the variability of our measurements due to leaf heterogeneity.

### Passive technique: sun-induced chlorophyll fluorescence

Passive measurements were made at both canopy and leaf scale. Canopy measurements were made with a field portable spectroradiometer (GER-1500,Geophysical & Environmental Research Corp., Milbrook, NY) operating at a spectral range between 350 and 1 050nm with a full width at half maximum (FWHM) of 3.2nm. At leaf level, a point spectroradiometer (ASD FieldSpec® 3, Analytical Spectral Devices, Boulder, CO, USA) coupled with the FluoWat leaf clip (Alonso *et al.*, 2007; Van Wittenberghe *et al.*, 2013) was used with a spectral range between 350 and 2500nm and a FWHM of 3 and 10nm in the 350–1050 and 1050–2500nm regions, respectively.

Sun-induced fluorescence (SIF) was measured using two passive approaches: indirect (FLD principle) and direct (short-pass filter). Measurements were made under natural illumination with clear sky conditions. The FLD principle retrieves the steady-state fluorescence emission from the solar irradiance and the radiance reflected and emitted by vegetation in certain solar irradiance absorption bands. The most important bands in the ChlF emission region, apart from the hydrogen absorption in the solar atmosphere (Hα, 656.4nm), are two of the oxygen absorption bands: OA at 763nm and OB at 687nm, both within a very narrow band (~2nm and ~1nm respectively). Importantly, at OA and OB absorption bands, ChlF is mainly emitted from PSI and PSII, respectively (Fig. 1A). Due to the GER-1 500 and ASD spectral resolution, in this experiment only the OA absorption band was used to calculate SIF. The OB absorption band is too narrow and the radiometric difference is too small to be detected by these instruments with FWHM 3.2 and 3.0nm. By comparing the depth of the absorption features of a solar irradiance with no fluorescence emission to the depth of a vegetation radiance with simultaneous measurements, it is possible to estimate the amount of SIF emitted by the plant and the actual reflectance (ρ) (Meroni *et al.*, 2009). The FLD basic concept has been upgraded with several modifications and improvements by different research groups (Stefan and Maier, 2003; Gomez-Chova *et al.*, 2006; Meroni and Colombo, 2006; Alonso *et al.*, 2008) to increase the accuracy of the methods and to exploit the current availability of high spectral resolution data (for a review of ChlF retrievals methods see Meroni *et al.*, 2009). In this study, the 3FLD763 technique, which uses a total of three bands (one inside and two outside the absorption band) to estimate SIF and ρ, was used to compute SIF at both canopy and leaf scale (Stefan and Maier, 2003) (Fig. 1B).

An alternative passive technique to measure vegetation fluorescence at leaf level consists of measuring the ChlF spectrum emission by coupling a spectroradiometer with the FluoWat leaf clip. Using this portable leaf clip, it is possible to measure the whole ChlF emission spectrum by cutting off the incoming light spectrum with a short-pass filter (<650nm). At wavelengths longer than 650nm, only the SIF emission is recorded, as light in this region is only emitted light. Though FluoWat allows measurement of the fluorescence emitted by both sides of the leaf, in this study only the upperside measurements were used. From the ChlF spectrum, SIF at 687nm and the area between 700 and 715nm (termed Fw687 and Fw700−715, respectively) were measured (Fig. 1B). By measuring at 687 and 700−715nm, we estimated the ChlF measured at OB and within the spectral range of the active technique, respectively. The FluoWat leaf clip was also used to measure the solar irradiance and leaf radiance needed to apply the FLD principle at leaf level. In addition, PAR was measured as the reflected radiance between 400 and 700nm of a spectralon panel (ODM-98, Gigahertz-Optik GmbH, Türkenfeld, Germany), before and after each canopy and leaf measurement. The emission of SIF is dependent on the intensity of the incoming PAR. Therefore, to be able to compare different leaf samples PAR measurements were used to compute the relative fluorescence yield ((SIFyield=SIF(λ)/PAR), where λ refers to the different wavelength at which SIF was measured (760nm at 3FLD760, 687nm at Fw687, and 700−715nm at Fw700−715).

Finally, the solar irradiance and leaf radiance measured with the Fluowat were used to compute the leaf reflectance (leaf reflectance=leaf radiance/solar irradiance). The leaf reflectance spectrum was used to estimate chlorophyll content by using the red edge R750 /R710 index (Zarco-Tejada *et al.*, 2001).

### Uncertainty

The coefficient of variation (CV) (=standard deviation/mean) of the main factors affecting leaf level ChlF measurements was computed for each day and treatment. The main factors driving CV were defined as (i) area of a single leaf, (ii) leaf heterogeneity, and (iii) leaf level measurements inputs. To avoid confusion, note an example of how the CV was computed for one of the factors taken into account in this study (i.e. the area of each single leaf): first, we computed the standard deviation and mean of each treatment and day; second, we computed the CV per treatment and day; finally we computed a unique CV per day [mean of CV for low (N1), medium (N3), and high nitrogen (N5) fertilizer per day].

Leaf area was estimated in the field by measuring the length and width of each single leaf. A bigger leaf area will lead to a higher ChlF re-mission and therefore changes in leaf area within replicates will increase the CV for ChlF measurements. Leaf heterogeneity is defined as chlorophyll content, stomatal conductance to water vapour and photosynthesis. Leaf heterogeneity could be associated with a broad number of factors, such as leaf thermal energy dissipation, leaf structure and leaf pigment distribution. In this study, we were able to perform simultaneous measurements of active and passive ChlF, chlorophyll content, stomatal conductance and CO2 assimilation. Leaves with higher chlorophyll content will lead to a higher ChlF re-mission; changes in chlorophyll content within replicates will increase the CV for ChlF measurements. Stomatal conductance constrains photosynthesis and thus variations in the efficiency of photosynthesis will modify the emission of ChlF. The measurement inputs of solar irradiance and leaf radiance also affect ChlF measurements. Incoming light drives photosynthesis and therefore also modulates fluorescence. In addition, these two parameters are the main inputs used to estimate ChlF using passive techniques. Therefore, any changes in solar irradiance and leaf radiance within replicates will also increase the CV for SIF measurements.

### Validation study: environmental controlled conditions

For the purpose of validating our results, we also computed the CV for leaf heterogeneity with active and passive ChlF measurements for a one-day experiment in cotton leaves growing under a controlled environment. Cotton plants were grown in a controlled-environment chamber at 25/18 °C with a 12h photoperiod and PAR of 500 μmol m^-2^ s^-1^ at the Arid-Land Agricultural Research Center in Maricopa, AZ. All plants were grown from seeds in 4-l pots containing a ready-made soil mixture (Sunshine mix#1, Sun Gro Horticulture, Canada). Plants were kept well-watered by adding a nutrient solution containing 2g l^-1^ of 20-20-20 Peters professional water solution fertilizer (Scotts-Sierra Horticultural Products Co., USA) and 0.5ml l^-1^ micronutrient solution of 2mM MnCl_2_, 10mM H_3_BO_3_, 0.4mM ZnSO_4_, 0.2mM CuSO_4_, 0.4mM Na_2_MoO_4_ and 0.1mM NiCl_2_, used at half-strength twice a week (Carmo-Silva and Salvucci, 2012).

After 20 d of plant growth, plants were moved to a greenhouse. Then after a further 8 d, measurements were taken outdoors over well-watered control plants and plants exposed to 7 d without watering. Measurements were taken on Day Seven from the beginning of the watering treatments. For this, 14 pots containing one plant each were divided into the two different treatments: well-watered and water-deficit, seven pots each. Two leaves per plant were measured (*n*=28).

### Statistical analysis

Statistical analyses were carried out using MATLAB 2011 (MathWorks Inc, Natick, USA) and Statistix v8.0 (Statistical Analytical, Tallahassee, USA). During the experiment 256 samples were collected; however after a detailed analysis of our dataset, 87 samples were eliminated due to bad spectroscopy measurements mostly at the low nitrogen samples. Our final dataset was reduced to 156 samples.

Differences between techniques in the different treatments were tested each day using one-way analysis of variance (ANOVA, Statistix v8.0, Statistical Analytical, Tallahassee, USA). Linear regressions of ChlF measured with F_s700−715_ and 3FLD_763_ techniques were fitted across all days to determine the overall relationship between active and passive measurements, using an F-test to test if slopes and intercepts were significantly different between days (linear regression, Statistix v8.0). If slopes were found to differ, pairwise slope comparisons were made using Tukey’s honestly significant difference (HSD) test, with HSD exceeding 3.34 considered significant (*P*≤0.05; Zar, 1974).

### Nomenclature

To avoid confusion in nomenclature, we refer to active measurements as F_s_ and passive measurements as SIF. To differentiate between canopy and leaf measurements, we add a ‘C’ or ‘L’ at the end of the active and passive term (SIFyield_C_ or SIFyield_L_ and ${\text{F}}_{s}$_L_ respectively). Additionally, we differentiate between single-leaf measurements (SIFyield_L_ and _${\text{F}}_{s}$L_) and average leaf measurements ($\bar{\text{SIFyield}}$_L_ and _$\bar{{\text{F}}_{s}}$L_). Finally, depending on the approach used to estimate ChlF, the active technique is referred as F_s700−715_ and three passive techniques are termed 3FLD_763_, Fw_687_ and Fw_700−715_ (Table 2).

**Table 2.** Definition and terminology of chlorophyll fluorescence measurements performed in this study. Photosynthetic active radiation (PAR) measurements were used to compute the relative sun-induced fluorescence (SIF) yield (SIF_yield_=SIF/PAR)

Spatial scale	Measurements	Terminology		Technique	Description
Canopy	Top of Canopy	Passive	SIFyield_C_	3FLD_763_	The whole plant measured from above the canopy
Leaf	Average	Active	$\bar{{\text{F}}_{s}}$_L_	F_s700−715_	A representative number of leaves measured then averaged to a unique value
		Passive	$\bar{\text{SIFyield}}$_L_	3FLD_763_ Fw_687_ Fw_700−715_	
Leaf	Single	Active	${\text{F}}_{s}$_L_	F_s700−715_	Individual leaves measured
		Passive	SIFyield_L_	3FLD_763_ Fw_687_ Fw_700−715_	

## Results and discussion

### Canopy scale

#### Seasonal measurements

**Fig. 2.**
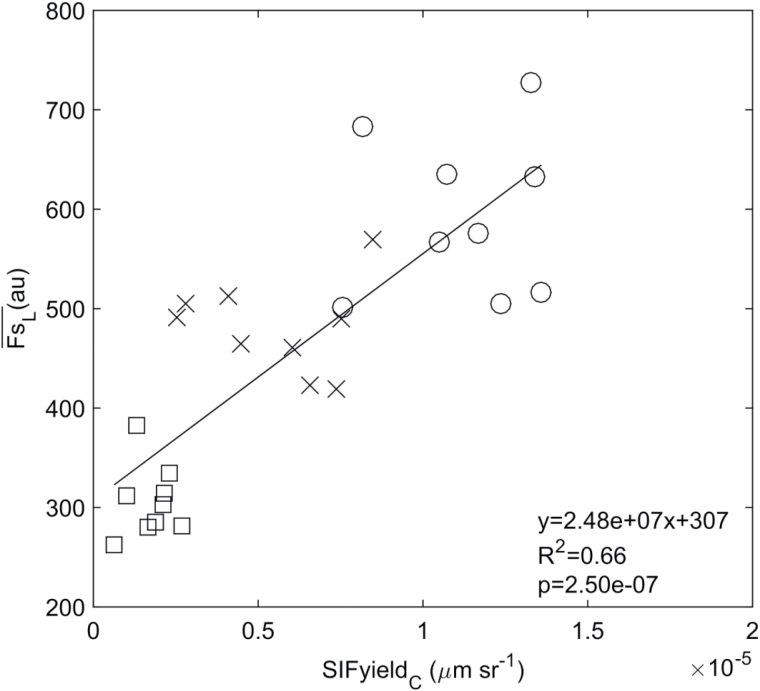
Canopy and leaf-average scale relationship between active leaf-average ($\bar{{\text{F}}_{s}}$_L_) and passive canopy (SIFyield_C_) measurements using *Fs_700−715_* and 3FLD_763_ techniques in wheat plants under low (square), medium (cross) and high fertilization treatment (circle). For leaf level measurements, each point represents the leaf replicates fluorescence mean (*n*=9) per day (days=9). The black line represents a regression between active and passive techniques (*P*<0.01).

As reported in previous studies (Zarco-Tejada *et al.*, 2003; Perez-Priego *et al.*, 2005; Rascher *et al.*, 2009), in order to compare active-leaf (${\text{F}}_{s}$_L_) and passive-canopy (SIFyield_C_) measurements a representative number of leaves were measured each day using the active technique and then averaged to a unique value ($\bar{{\text{F}}_{s}}$_L_). When compared with the SIFyield_C_ measurement using the 3FLD_763_ technique, a significant positive linear relationship was observed between $\bar{{\text{F}}_{s}}$_L_ and SIFyield_C_ (*R* ^2^=0.64 and *P*<0.01) for the whole season across treatments (Fig. 2).

In this study, the red edge index provided the best approach to discriminate between nitrogen treatments (See Supplementary Table S1 at *JBX* online); and chlorophyll fluorescence changes were driven by variations in leaf chlorophyll content in response to nitrogen deficit (See Supplementary Fig. S1 at *JBX* online). As described in the introduction, chlorophyll fluorescence is not simply a measure of chlorophyll content, it also provides information about plant photosynthetic capacity. Previous studies have shown that the decrease in photosynthesis modulated chlorophyll fluorescence via different mechanisms depending on the treatment: through the action of NPQ in response to water stress, or through the action of changes in leaf chlorophyll concentration in response to nitrogen deficiency (Cendrero-Mateo *et al.*, 2015). Additionally, Rascher *et al.* (2015) showed that the correlation between canopy chlorophyll content and chlorophyll fluorescence measurements presented a clear deviation from a simple 1:1 line when different species under different functional status were plotted together.

The result presented in this paper corroborates studies by Perez-Priego *et al.* (2005) and Rascher *et al.* (2009) and suggests that when nitrogen drives changes in ChlF, $\bar{{\text{F}}_{s}}$_L_ measurements can be used to better understand canopy measurements of SIF. Nevertheless, a set of factors and phenomena needs to be taken into account when linking leaf active measurements and canopy passive measurements. The canopy SIF signal (SIFyield_C_) reaching the sensor is different from the SIF signal emitted by the leaf (SIFyield_L_). The leaf fluorescence signal will change its spectral properties as it travels through the canopy, due to the variable and selective reabsorption of fluorescence by chlorophyll within the canopy (Gitelson *et al.*, 1998; Franck *et al.*, 2002).

### Leaf-average scale

#### Seasonal measurements

**Fig. 3.**
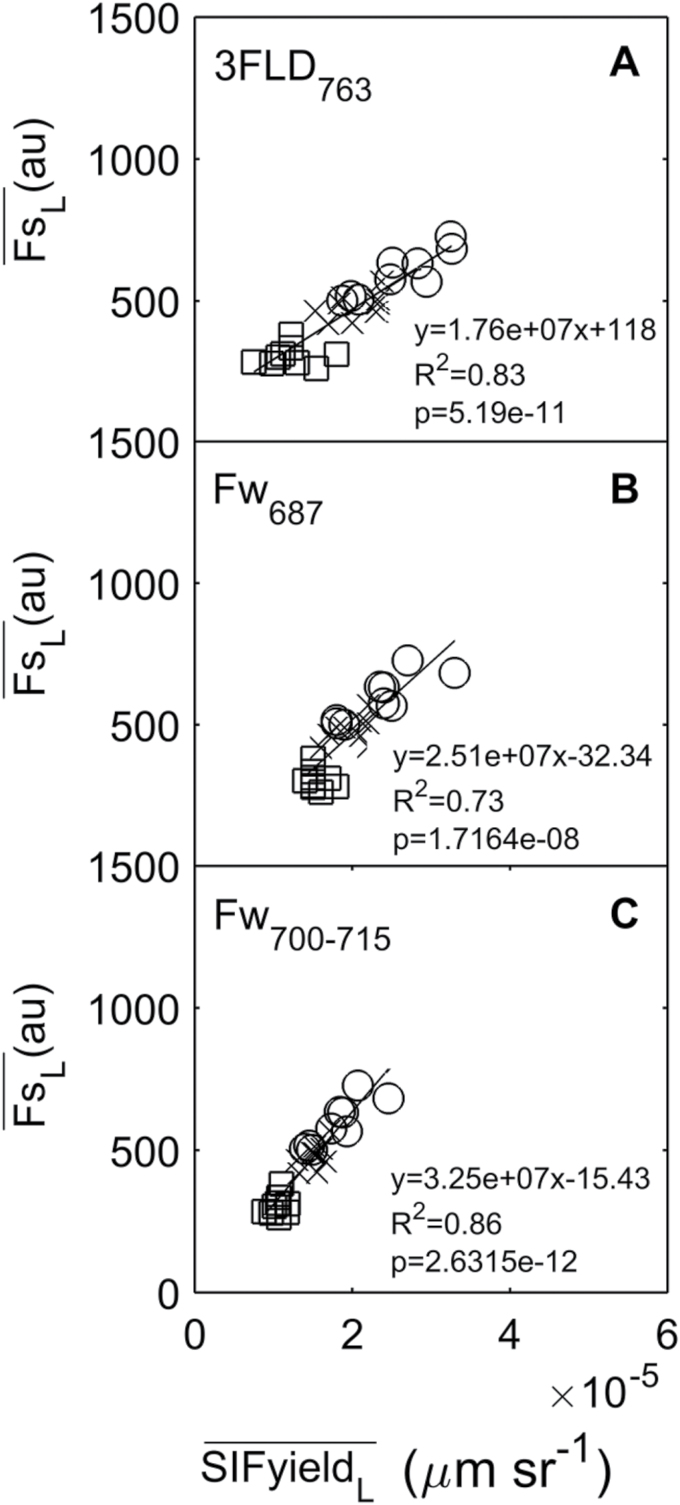
Leaf-average scale relationship between active (Fs_L_) and passive **(SIFyield**_L_) measurement, using (A) F_s700**−**715_ and 3FLD_763_ techniques, (B) F_s700**−**715_ and Fw_687_ techniques, and (C) F_s700**−**715_ and Fw_700**−**715_ techniques in wheat plants under low (square), medium (cross) and high (circle) fertilization treatment. Each point represents the leaf replicates fluorescence mean (*n*=9) per day (days=9). The black line represents a regression between active and passive techniques (*P*<0.01).

A daily average of the ChlF for each technique and nitrogen treatment was computed (Fig. 3). A significant positive linear relationship was observed between active and all passive techniques across treatments (*R* ^2^≥0.73 and *P*<0.01). However, a constant bias between techniques was observed, and contrary to the results of Moya *et al.* (2004) where light was the limiting factor, no zero intercept was found. Most likely the important differences between both measuring principles get exacerbated when light is not the limiting factor. These are (i) the wavelengths at which fluorescence is measured and (ii) the wavelengths and intensity used to excite fluorescence.

That is, PAM fluorometers measure ChlF between 700 and 715nm and in contrast the FLD principle measures ChlF in a narrower band, 3FLD_763_ or Fw_687_ (Fig. 1; Table 1). Even if ChlF had been measured at the same wavelength (e.g. using the F_s700−715_ and Fw_700−715_ techniques), it is known that changes in excitation wavelength and light intensity produce differences in the fluorescence emission (Corp *et al.*, 2003; Louis *et al.*, 2006). For the active technique, fluorescence was excited by a very brief but strong light, whereas for the passive technique fluorescence was continuously excited by sunlight (Table 1).

$\bar{\text{SIFyield}}$_L_ measured with the 3FLD_763_ and Fw_700−715_ techniques presented stronger correlation with $\bar{{\text{F}}_{s}}$_L_ than when measured with the Fw_687_ technique (*R* ^2^=0.83, 0.86 and 0.73, respectively) (Fig. 3). Additionally, ChlF measured with 3FLD_763_ and F_s700−715_ techniques presented the same results in terms of differentiating between nitrogen treatments at the seasonal scale; where low, medium and high nitrogen were statistically different (Table 3). $\bar{\text{SIFyield}}$_L_ measured with the Fw_687_ technique was not able to discriminate between low, medium and high nitrogen treatments (Table 3). ChlF measured at 687nm is less sensitive to change in nitrogen concentration due to the partial reabsorption of the emitted red fluorescence by the absorption band of the leaf chlorophyll. ChlF at 700−715nm and 763nm is much less affected by re-absorption and therefore is used as a better indicator of nitrogen deficit (Buschmann, 2007; Konanz *et al.*, 2014).

**Table 3.** Results of the repeated-measures ANOVA F-test comparing effects of nitrogen treatment (L, low; M, medium; H, high) on leaf-average fluorescence measurements between active (F_s700−715_) and passive (Fw_687_, 3FLD_763_, and Fw_700−715_) techniques. Passive techniques are expressed as sun-induced fluorescence yield (SIF_yield_=SIF/PAR). Bold figures indicate that active and passive techniques provided the same results; grey columns indicate the days when this occurred. Superscripts a, b, c, denote significant differences at *P*<0.05 (ANO**VA**) for low nitrogen, medium nitrogen and high nitrogen treatments, respectively

Days											
Technique	N	All	55	62	69	83	90	97	104	111	118
**F_s700−715_**	**L**	**308^a^**	282^a^	312^a^	262^a^	**381^a^**	**334^a^**	**303^a^**	314^a^	**279^a^**	**285^a^**
	**M**	**474^b^**	422^b^	504^b^	513^b^	**464^a-b^**	**490^b^**	**460^b^**	568^b^	**419^b^**	**490^b^**
	**H**	**590^c^**	575^c^	567^b^	682^c^	****501^b^****	**505^b^**	**516^b^**	727^c^	**631^c^**	**634^c^**
***3FLD_763__(×10_^–5^_)_***	**L**	**1.18^a^**	1.30^a^	1.80^a^	1.54^a^	1.22^a^	**1.22^a^**	**1.06^a^**	1.12^a^	**0.98^a^**	**0.74^a^**
	**M**	**2.03^b^**	2.00^a-b^	2.32^a-b^	1.87^a^	1.53^a^	**2.32^b^**	**2.30^b^**	2.37^b^	**1.69^b^**	**1.83^b^**
	**H**	**2.58^c^**	2.48^b^	2.94 ^b^	3.26 ^b^	1.87^a^	**2.08^b^**	**1.98^b^**	3.25^b^	**2.83^c^**	**2.51^c^**
***Fw_687__(×10_^–5^_)_***	**L**	1.56^a^	1.77^a^	1.69^a^	1.65^a^	1.48^a^	1.50^a^	1.44^a^	1.54^a^	1.50^a^	1.48^a^
	**M**	1.97^a-b^	2.18^a^	2.19^a^	2.10^a-b^	1.68^a^	1.80^a^	2.07^a-b^	2.17^a-b^	1.61^a^	1.92^a-b^
	**H**	2.36^b^	2.43^a^	2.53^a^	3.33^b^	1.93^a^	1.84^a^	1.83^b^	2.66^b^	2.40^b^	2.35^b^
**Fw_700−715__(×10_^–5^**_)_	**L**	1.57^a^	1.17^a^	1.18^a^	1.07^a^	**1.10^a^**	**1.07^a^**	**1.01^a^**	1.05^a^	0.97^a^	0.88^a^
	**M**	2.28^b^	1.55^a-b^	1.61^a-b^	1.52^a^	**1.30^a-b^**	**1.52^b^**	**1.66^b^**	1.71^b^	1.32^a^	1.49^b^
	**H**	2.71^b^	1.73^b^	1.93 ^b^	2.46^b^	**1.49 ^b^**	**1.40^b^**	**1.45^b^**	2.07^b^	1.88^b^	1.84^b^

#### Daily measurements

A daily-scale ANOVA F-test comparing effects of nitrogen treatment on leaf-average ChlF measurements between active and passive techniques was computed (Table 3). Similar to results for the seasonal scale, $\bar{\text{SIFyield}}$_L_ measured with the 3FLD_763_ and Fw_700−715_ techniques showed better agreement with $\bar{{\text{F}}_{s}}$_L_ than with Fw_678_ at the daily scale in terms of differentiating between nitrogen treatments (Table 3, shaded columns). In contrast, the results obtained using the Fw_687_ technique mostly mismatched the results presented by the active technique and the 3FLD_763_ and Fw_700−715_ techniques.

**Fig. 4.**
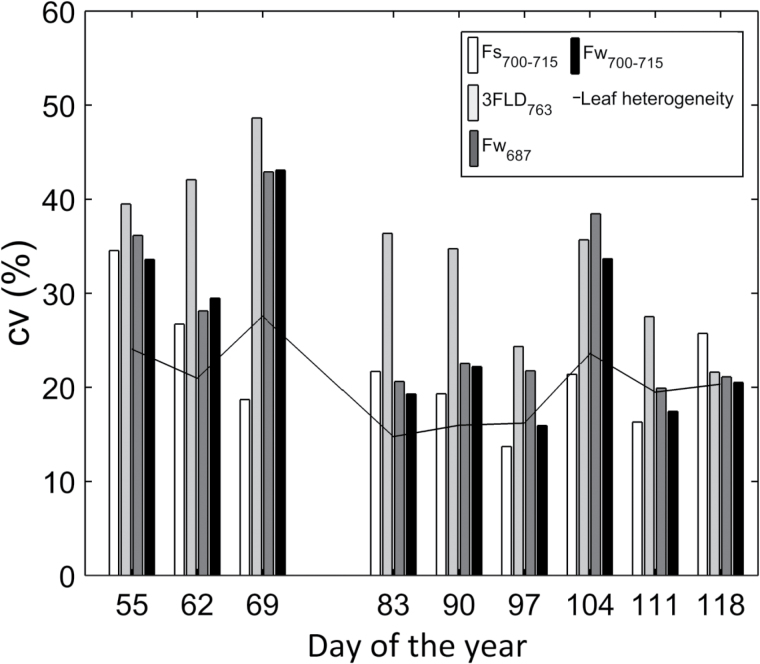
Coefficients of variation (CV) of leaf-scale active ChlF measurements (using the *F_s700−715_* technique – white bar), passive *ChlF* measurements (using the *3FLD_763_*, *Fw_687_*, and *Fw_700-715_* techniques – light grey, dark grey, and black bar respectively), and leaf heterogeneity (chlorophyll content, photosynthesis and stomatal conductance), line, by day of year. Each bar represents the average CV for combined low, medium and high nitrogen treatments.

It is important to note that later in the season the variability in leaf heterogeneity decreased (days 83, 90, 97, 111 and 118: Fig. 4, black line). This corresponded with the days when ChlF measured with the F_s700−715_, 3FLD_763_ and Fw_700−715_ techniques showed the same response to the nitrogen treatments (Fig. 4; Table 3). In contrast, for the days with higher variability for leaf heterogeneity (days 55, 62, 69 and 104: Fig. 4), no match was found between techniques (Table 3). The results obtained at the daily scale supported the results shown at the seasonal scale (Fig. 3). $\bar{\text{SIFyield}}$_L_ measured with the Fw_687_ technique also exhibited less sensitivity to changes in nitrogen treatments (Table 3).

### Single-leaf scale

#### Seasonal measurements

**Fig. 5.**
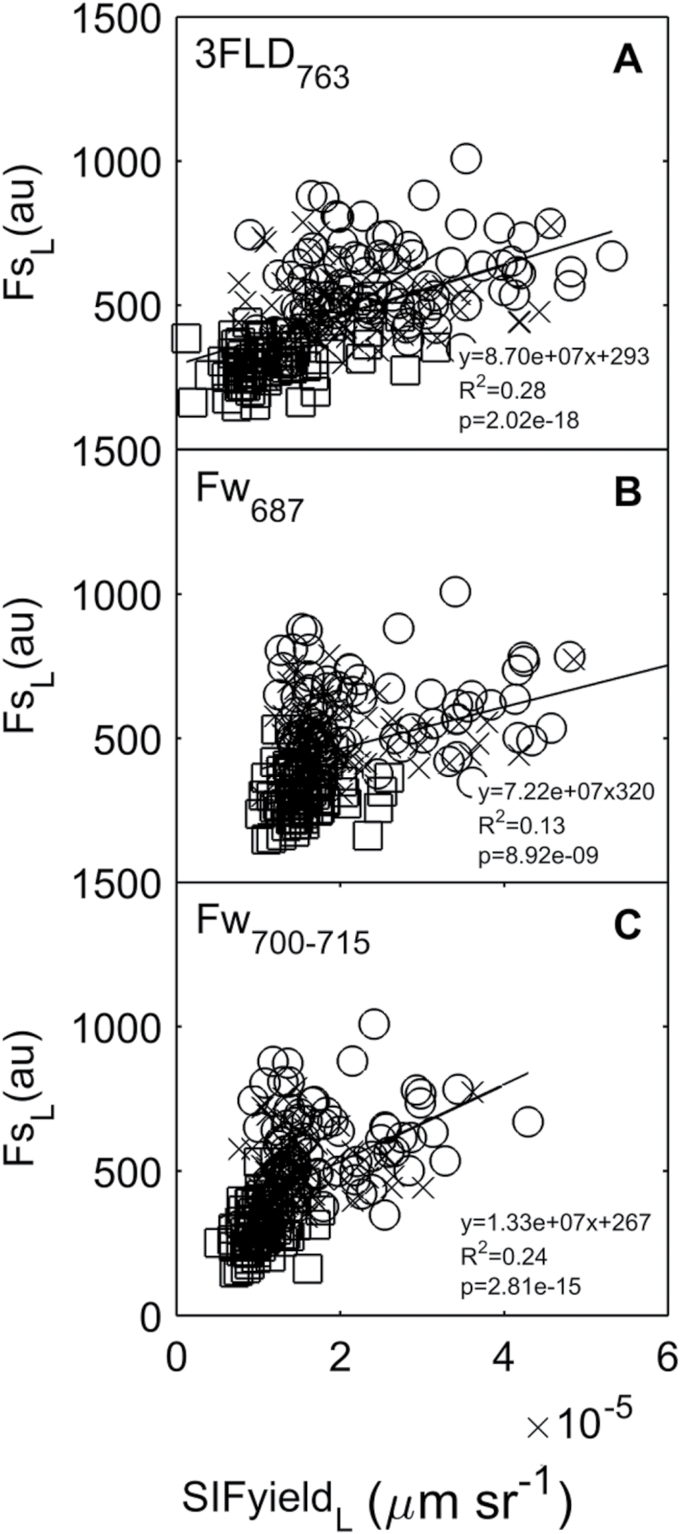
The leaf-scale relationship between active (FsL) and passive (SIFyield_L_) measurement using (A) F_s700−715_ and 3FLD_763_ techniques, (B) F_s700−715_ and Fw_687_ techniques, and (C) F_s700−715_ and Fw_700−715_ techniques in wheat plants under low (square), medium (cross) and high (circle) fertilization treatment. The black line represents a regression between ChlF measurements with active and passive techniques (*n*=156, *P*<0.01).

At the single-leaf scale, a weak but significant relationship was observed between active and passive techniques across treatments (Fig. 5). SIFyield_L_ values measured with the three passive techniques were less sensitive to changes in ChlF when _${\text{F}}_{s}$L_ reached 500 au. The coefficient of determination (*R* ^2^) between active and passive techniques increased by 30%, 4% and 30% using the 3FLD_763_, Fw_687_ and Fw_700−715_ techniques, respectively, when values of ${\text{F}}_{s}$_L_>500 were not taken into account in the study.

Similar to leaf-average results, SIFyield_L_ measured with 3FLD_763_ and F_w700−715_ techniques offered the best correlations with ${\text{F}}_{s}$_L_ (*R* ^2^=0.28 and 0.24, respectively, *P*<0.001: Fig. 5A, C). Since only the 3FLD_763_ technique can be applied using satellite remote sensing techniques, results presented from here on will focus on this passive technique.

**Fig. 6.**
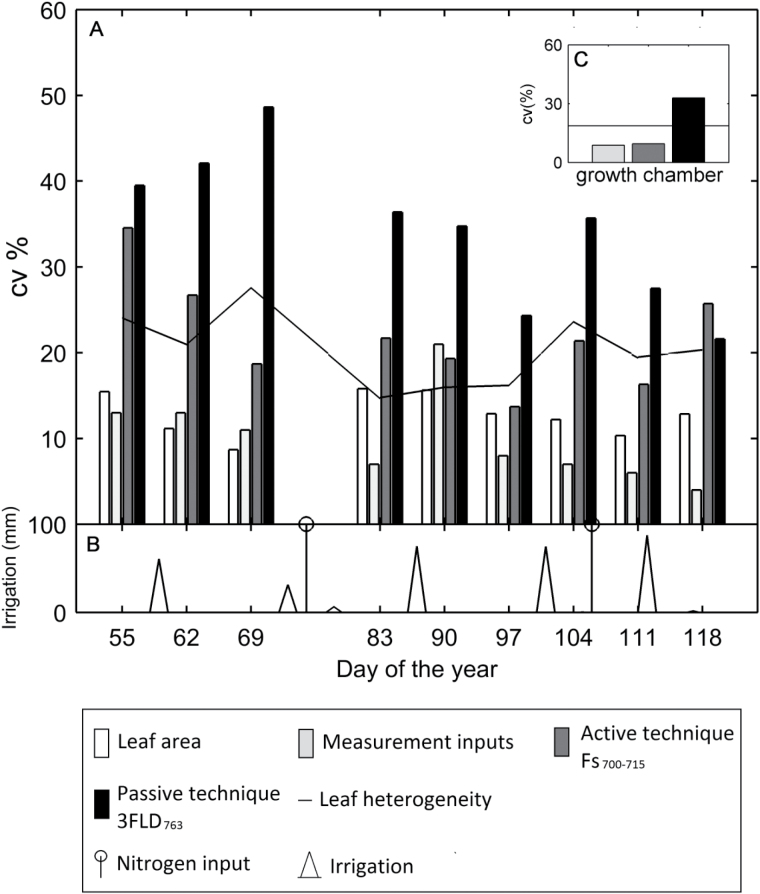
(A) Coefficient of variation (CV) of the main factors affecting chlorophyll fluorescence measurements: leaf area, white bar; measurement inputs (PAR, solar irradiance and vegetation radiance), light grey bar; and leaf heterogeneity (chlorophyll content, photosynthesis and stomatal conductance), line. Also included is the CV for active (F_s700−715_, dark grey bar) and passive (3FLD_763_, black bar) ChlF measurement techniques. (B) Irrigation (triangle) and nitrogen input (circle and line). (C) Coefficient of variation under control conditions for leaf heterogeneity (line), measurement inputs (light grey bar), ChlF based on the active technique (F_s700−715_, dark grey bar), and the passive technique (3FLD_763_, black bar).

**Fig. 7.**
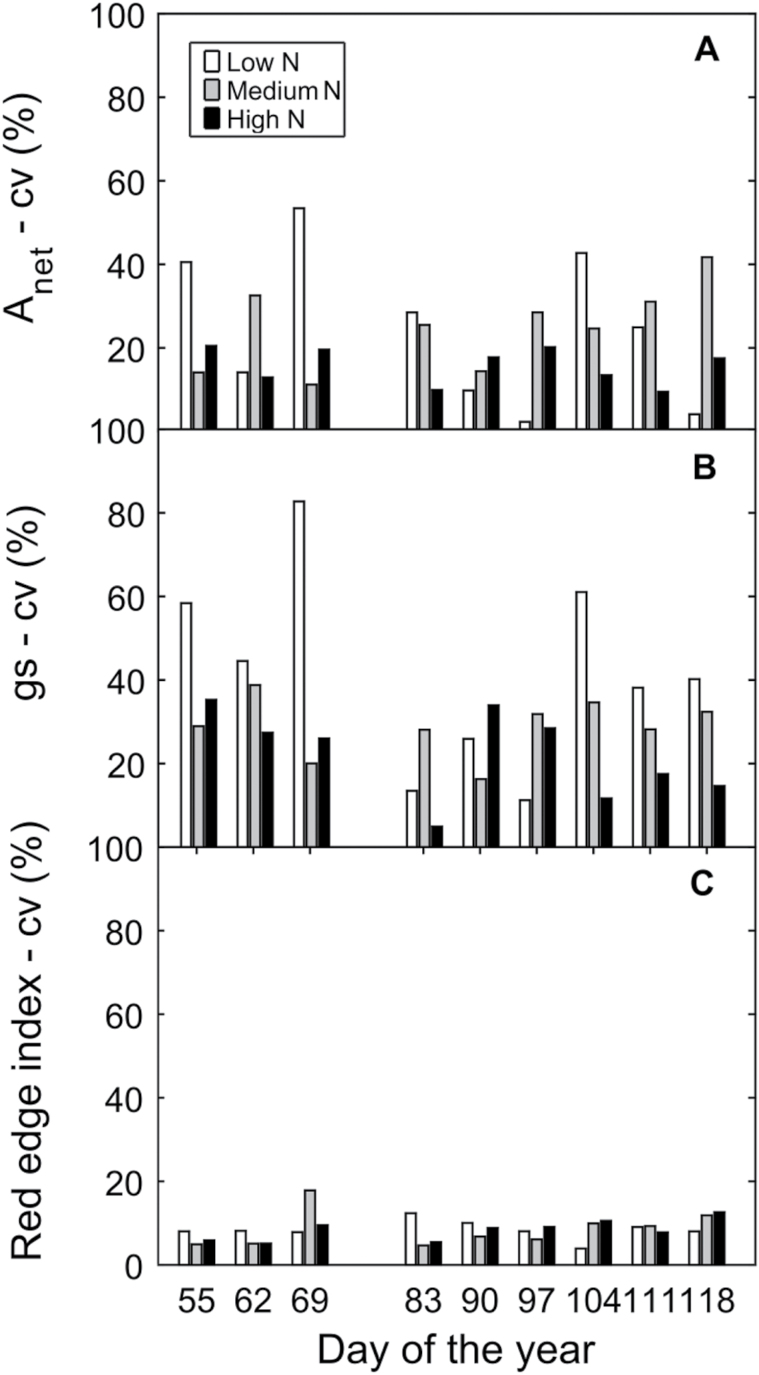
Coefficient of variation (CV) of the main factors affecting leaf heterogeneity: (A) photosynthesis, (B) stomatal conductance, and (C) chlorophyll content estimated using the red edge vegetation index.

We found a large scatter by analysing a leaf-to-leaf correlation between techniques (Fig. 5). To understand what is causing this dispersion, we compared the variation of the main factors affecting ChlF measurements − leaf area, leaf heterogeneity and measurements inputs – with variation of active (${\text{F}}_{s}$_L_) and passive (SIFyield_L_) ChlF measurements (Figs 6, 7). Leaf area and measurement inputs presented a low CV for all the days (<20%, Fig. 6A). The fact that the measurement inputs presented a low CV indicate that the scattering observed was not due to the measurement protocol. That is, on different days, similar leaf spectrum and illumination conditions were observed within treatments. In contrast, the parameters associated with leaf-to-leaf variability (leaf stomatal conductance, leaf photosynthesis and leaf chlorophyll content) presented a higher CV (15%<C<80%) which changed through time (Fig. 7). Importantly, stomatal conductance and leaf photosynthesis presented a higher CV (15%<CV<80%, Fig. 7A, B) than chlorophyll content (CV<20%, Fig. 7C). As described before, on the days when leaf heterogeneity had lower CV SIFyield_L_ and ${\text{F}}_{s}$_L_ measurements also had low CV (days 83, 90 and 97). However, the CV for SIFyield_L_ measured with the 3FLD_763_ technique was consistently higher than for ${\text{F}}_{s}$_L_ using the F_s700−715_ technique. Additionally, irrigation and fertilizer input did not seem to affect the CV of leaf heterogeneity or $\bar{\text{SIFyield}}$_L_ and ${\text{F}}_{s}$_L_ measurements (Fig. 6B).

These results imply that the scattering observed in our measurements is mainly due to the leaf-to-leaf variability. To corroborate our results, we compared the variations in wheat (grown outdoors) with those in the cotton experiment (grown in a growth chamber, Fig. 6C). For the cotton experiment, the CV for leaf heterogeneity, SIFyield_L_ and ${\text{F}}_{s}$_L_ were 27%, 57% and 4% lower, respectively, than the seasonal average CV for those variables in the wheat experiment. Notably, the CV for *$\bar{\text{SIFyield}}$*_L_ using the 3FLD_763_ technique was again higher than the CV for ${\text{F}}_{s}$_L_. These results confirmed that the outdoor field measurements increased leaf-to-leaf variability. It is probably due to the rapidly reversible quenching, NPQ, which modulates ChlF. NPQ is limited by the intrinsic capacity of each leaf to dissipate excess light as heat (Serôdio and Lavaud, 2011). Still, the passive ChlF measurements were consistently more variable (higher CV) than leaf heterogeneity and active ChlF measurements for both growth chamber and outdoor experiments. These results make evident the complexity of measuring ChlF using indirect passive techniques as compared with active techniques. However, it is important to note that PAM fluorimeters have a limited application to spatial scales ranging from several centimetres to some metres. In contrast, passive techniques can be applied at leaf and canopy scales, as well as from regional to global scales.

#### Daily measurements

**Fig. 8.**
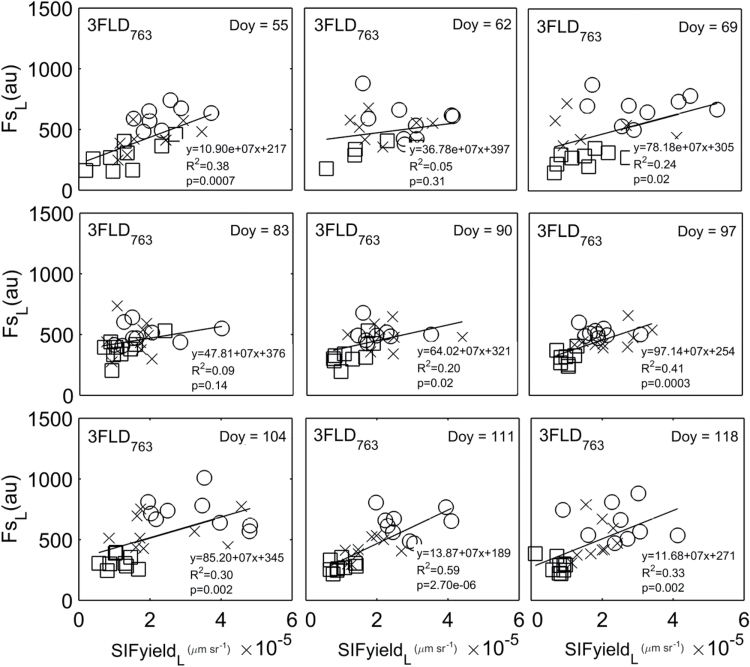
Leaf-scale relationship per day between active (${\text{F}}_{s}$_L_, measured using F_s700−715_) and passive measurement (SIFyield_L_, measured using 3FLD_763_), in wheat plants under low (square), medium (cross) and high fertilization treatment (circle). Each point represents one leaf measurement (n=9) per day (n=9). The black line represents a regression between ChlF measurements based on active and passive techniques (P<0.01). Results in bold font, P<0.05.

A weak but significant relationship was observed between SIFyield_L_ and ${\text{F}}_{s}$_L_ across treatments at the leaf scale for most of the days (*P*≤0.05, days 55, 69, 90, 97,104,111, 118: Fig. 8). The best correlation between techniques (here F_s700−715_ and 3FLD_763_) was found toward the end of the season (day 111, *R* ^2^=0.59, *P*<0.01), when both techniques were also able to differentiate between the three nitrogen treatments at leaf-average measurements (Table 3). The accumulated nitrogen stress resulted in lower chlorophyll content in the leaves in low and medium treatments causing lower ChlF. By day 111, substantive leaf yellowing was observed in the low nitrogen treatment.

Pairwise comparison of slopes showed no significant difference between day 55 and 111, day 55 and 118, day 69 and 90, day 69 and 97 (*P*<0.05). These results showed that for this experiment at the leaf spatial scale and the daily temporal scale, it was not possible to define a unique equation to relate SIF to active ChlF measurements.

## Conclusions

This paper presents a study of the correlation between active and passive techniques to measure chlorophyll fluorescence at canopy and leaf scale for wheat plants under different nitrogen treatments. The results presented in this study showed that passive and active measurements were highly correlated over the growing season across nitrogen treatments at both canopy and leaf-average scale. However, a constant bias between techniques was observed and no zero intercept was found. This was likely due to their different physical measuring principles regarding the wavelength at which fluorescence is measured and the wavelength and intensity used to excite fluorescence. For leaf-average measurements, the ChlF measured with the passive 3FLD_763_ and Fw_700−715_ techniques presented the strongest agreement (*R* ^2^>0.7, *P*<0.01) in terms of differentiating between nitrogen treatments at both the seasonal and daily scale. At the leaf scale, the seasonal relationship between passive and active measurements was weaker, but still significant, where leaf-scale ChlF measured with the 3FLD_763_ technique showed the best correlation with ChlF measured with the F_s700−715_ technique. The sources of uncertainty at the leaf scale were largely related to leaf-to-leaf variability associated with spatial and seasonal variations in CO_2_ assimilation and stomatal conductance, and less related to the leaf size or measurement inputs (e.g. light reflected and emitted by the leaf and illumination conditions). This uncertainty was exacerbated when the analysis was limited to the leaf scale on a single day, where our results showed that it was not possible to define a unique equation to relate SIF to active ChlF measurements.

Based on these findings, we conclude that it is possible to compare canopy and leaf-average measurements of active and passive techniques at both daily and seasonal temporal scales when nitrogen is the limiting factor. By averaging a number of representative leaves to a unique value, we reduced the variability between measurements due to (i) different physical measuring principles between techniques and (ii) leaf-to-leaf heterogeneity.

Nevertheless, to extrapolate the knowledge acquired using active techniques to passive techniques, it will first be necessary to quantify how the two factors mentioned above affect ChlF measurements. Second, these findings should be incorporated into a radiative transfer model where the vegetation structure is taken into account.

These steps are particularly relevant to the interpretation of ChlF data when the signal is obtained in different spectral regions that will consequently carry information from different layers of the leaf or the canopy (Porcar *et al.* 2014). At leaf and canopy levels, the red ChlF signal is enriched in photosystems close to the leaf surface or leaves from the top of the canopy, whereas the far-red ChlF signal may have a stronger contribution from a deeper leaf or canopy layer, especially when the excitation light penetrates deep into the leaf or the canopy (Peterson *et al.*, 2001; Rappaport *et al.*, 2007; Pfündel, 2009). When scaling up from the leaf to canopy level, the bidirectional ChlF emission (both upward and downward) as well as vegetation structure (for multiple-scattering and re-absorption) needs to be modelled (Van Wittenberghe *et al.*, 2015). The orientation of the leaves and the incident light angle will also play an important role in ChlF emission.

For a quantitative analysis of how ChlF measurements are affected by the wavelength at which fluorescence is measured and by the wavelength and intensity used to excite fluorescence, dedicated leaf-scale studies should be designed. In these experiments a fluorescence excitation matrix should be created (FluorMOD, Pedrós *et al.*, 2010). That is, high spectral resolution measurements of ChlF should be performed at the same wavelength and intensity used to excite fluorescence changes. This will enable a good understanding of how the ChlF spectrum changes depending on the spectral properties of the incoming light. Additionally, to account for the ChlF spatial and temporal dynamics, these measurements should be repeated in several leaves under different stress conditions (i.e. water or nitrogen deficit) as well as at different vegetation stages. This can be used to better estimate plant photosynthetic capacity and therefore to provide improved information for crop management.

## Supplementary data

Supplementary data is available at *JXB* online.

Supplementary Figure S1. The relationship between canopy chlorophyll content (red edge index) and passive fluorescence (3FLD_763_) in wheat plants under low, medium and high fertilization treatment.

Supplementary Table S1. Results of the repeated-measures ANOVA F-test comparing effects of nitrogen treatment on canopy chlorophyll content (red edge index) and passive fluorescence (3FLD_763_).

Supplementary Data
