# Supplementary material for: Plant chlorophyll fluorescence: active and passive measurements at canopy and leaf scales with different nitrogen treatments
Source: J Exp Bot. 2015 Oct 19;67(1):275–86. doi: 10.1093/jxb/erv456 (PMC4682433; doi:10.1093/jxb/erv456)
Supplement: Supplementary Data [file supp_67_1_275__index.html]

Plant chlorophyll fluorescence: active and passive measurements at canopy and leaf scales with different nitrogen treatments — Plant chlorophyll fluorescence: active and passive measurements at canopy and leaf scales with different nitrogen treatments — Supplementary Data 

# Plant chlorophyll fluorescence: active and passive measurements at canopy and leaf scales with different nitrogen treatments

## Supplementary Data

Data files

- Supplementary Data - Supplementary Data
