# Supplementary material for: Plant chlorophyll fluorescence: active and passive measurements at canopy and leaf scales with different nitrogen treatments
Source: J Exp Bot. 2015 Oct 19;67(1):275–86. doi: 10.1093/jxb/erv456 (PMC4682433; doi:10.1093/jxb/erv456)
Supplement: Supplementary Data [file supp_erv456_supplementary_data.pdf]

Supplementary data

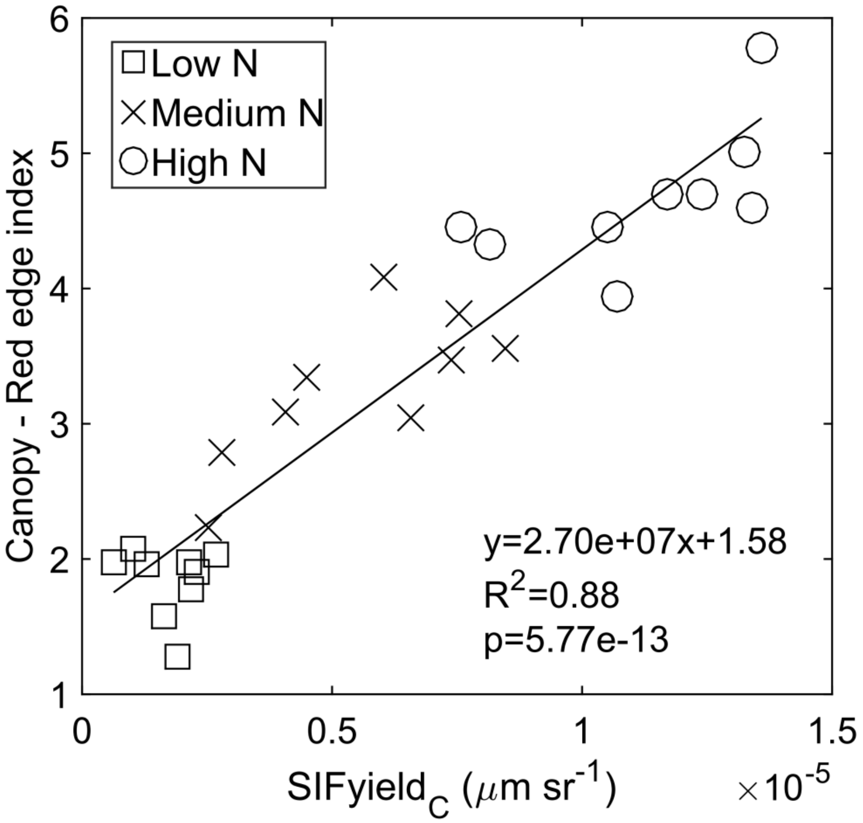

Supplementary Figure S1. Canopy chlorophyll content (red edge index) and passive fluorescence ($3FLD_{763}$) relationship in wheat plants under low (square), medium (cross), and high fertilization treatment (circle). For canopy level measurements, each point represents chlorophyll content and passive fluorescence measurements per day (number days = 9). The black line represents a regression between chlorophyll content and passive fluorescence ($p<0.01$).

Supplementary Table S1. Results of the repeated-measures ANOVA F-test comparing effects of nitrogen treatment (low (L), medium (M) and High (H)) on canopy chlorophyll content (red edge index) and passive fluorescence ($3FLD_{763}$). Passive fluorescence is expressed as Sun Induced Fluorescence yield ($SIF_{yield} = SIF/PAR$). Bolded values indicate that chlorophyll content and passive fluorescence provided the same results. Columns with grey shading highlight the days when that happened ($p < 0.05$).

			Days								
**Technique**	**N**	**All**	**55**	**62**	**69**	**83**	**90**	**97**	**104**	**111**	**118**
Red-Edge	**L**	**1.83**^{a}	**2.03**^{a}	2.07^{a}	1.96^{a}	1.96^{a}	**1.91**^{a}	**1.97**^{a}	**1.77**^{a}	**1.56**^{a}	1.27^{a}
	**M**	**3.26**^{b}	**3.04**^{b}	2.78^{b}	3.08^{b}	3.34^{b}	**3.81**^{b}	**4.08**^{b}	**3.55**^{b}	**3.47**^{b}	2.22^{b}
	**H**	**4.66**^{c}	**4.68**^{c}	4.45^{c}	4.31^{c}	4.45^{c}	**4.70**^{c}	**5.78**^{c}	**5.01**^{c}	**4.61**^{c}	3.94^{c}
$3FLD_{763}$ ($x10^{-6}$)	**L**	**0.25**^{a}	**1.35**^{a}	0.64^{a}	0.63^{a}	1.30^{a}	**1.91**^{a}	**2.13**^{a}	**2.16**^{a}	**1.64**^{a}	1.90^{a}
	**M**	**5.34**^{b}	**5.31**^{b}	2.29^{a}	4.08^{a}	4.49^{a-b}	**7.53**^{b}	**6.02**^{b}	**8.47**^{b}	**7.38**^{b}	2.52^{a}
	**H**	**11.3**^{c}	**11.7**^{c}	10.1^{b}	8.17^{b}	7.57^{b}	**12.4**^{c}	**13.6**^{c}	**13.3**^{c}	**13.4**^{c}	10.7^{b}
